# Influence of different airway devices on intra-arrest ventilation during bag-valve-device ventilation - a prospective randomized controlled cadaver study

**DOI:** 10.1186/s13054-025-05713-z

**Published:** 2025-12-05

**Authors:** Jonas Lohmann, Beate Brand-Saberi, Mahsa Ullrich, Tamar Gelashvili, Annika Hoyer, Lydia Johnson Kolaparambil Varghese, Vanessa Kuehn, Christian Neuhaus, Claudia Schneider, Justin Trenkel, Jochen Hinkelbein, Gerrit Jansen

**Affiliations:** 1 Medical School OWL, Bielefeld University, Universitätsstraße 25, 33615 Bielefeld, Germany; 2 Department of Anatomy and Molecular Embryology, Institute of Anatomy, Medical Faculty of Ruhr University Bochum, Ruhr University Bochum, Universitätstraße 150, 44801 Bochum, Germany; 3 WEINMANN Emergency Medical Technology GmbH + Co. KG, Frohbösestraße 12, 22525 Hamburg, Germany; 4 University Department of Anesthesiology, Intensive Care Medicine, Emergency Medicine and Pain Medicine, Johannes Wesling Klinikum Minden, Ruhr University Bochum, Hans- Nolte-Straße 1, 32429 Minden, Germany; 5 Medical School OWL, Biostatistics and Medical Biometry, Bielefeld University, Universitätsstraße 25, 33615 Bielefeld, Germany

**Keywords:** Resuscitation, Airway management, Ventilation, artificial, Advanced Cardiac Life Support

## Abstract

**Background:**

Out-of-hospital cardiac arrest remains a major challenge due to its high incidence and low survival rates. In recent decades, research has focused on the performance of chest compressions and improvements in early defibrillation, while the optimal ventilation strategy remains unclear. Despite the lack of monitoring systems, manual bag-valve-device ventilation is still common. Given the potential impact of both the applied volumes and the ventilation pressures on hemodynamics and resuscitation efforts, the present study investigated the effects of various airway devices on the target parameters of ventilation therapy during manual intra-arrest ventilation.

**Methods:**

Thiel-embalmed human cadavers were included in a standardized resuscitation scenario. Ventilation was performed in a randomized order using various airway devices (tracheal tube (ET), supraglottic airway devices (SGA): laryngeal mask airway, laryngeal tube, i-gel^®^ laryngeal mask) and manual bag-valve-device ventilation. Chest compressions were performed via Corpuls-CPR. Ideal (Vt_ideal_), expiratory (Vt_e_) and inspiratory (Vt_i_) tidal volumes; leakage volume (V_leak_); and peak (P_peak_) and mean (P_mean_) pressures were recorded. The primary efficacy endpoint was the difference between Vt_ideal_ and Vt_e_ (∆Vt).

**Results:**

Eleven cadavers were included (mean age at the time of death: 84 ± 3.7 years, male = 7 [63.6%]). During ventilation with ET, the following mean values were measured: ΔVt, 142.2 ± 125.5 ml; Vt_e,_ 245.1 ± 91.2 ml; V_leak,_ 17.9 ± 15.3%; P_mean,_ 4.0 ± 1.5 mbar; and P_peak,_ 47.7 ± 14.9 mbar. During ventilation with SGA, however, the mean values were ΔVt, 202.0 ± 153.1 ml; Vt_e,_ 183.8 ± 122.1 ml; V_leak,_ 39.0 ± 23.5%; P_mean,_ 3.1 ± 1.0 mbar; and P_peak,_ 39.0 ± 10.0 mbar. Comparison of the two groups revealed lower ΔVt values (regression coefficient [RC] = –61.07 ml, 95% confidence interval [95% CI] = [–93.58;–28.55], p = 0.0003) and V_leak_ values (RC = –20.98%, 95% CI = [–26.63;–15.33], p<0.0001), but higher Vt_e_ values (RC = 61.14 ml, 95% CI = [28.63;93.65], p = 0.0003), P_mean_ values (RC = 0.90 mbar, 95% CI = [0.59;1.21], p<0.0001), and P_peak_ values (RC = 11.46 mbar, 95% CI = [8.65;14.26], p<0.0001) in the ET group. There was no evidence for differences in ΔVt among the cadavers in the SGA group.

**Conclusion:**

The ∆Vt was lower in the ET group than the SGA group, whereas there was no evidence for differences in ∆Vt among the devices in the SGA group.

**Trial registration:**

clinicaltrials.gov; Unique identifier: NCT06306898, Registration date: 5 March 2024.

**Supplementary Information:**

The online version contains supplementary material available at 10.1186/s13054-025-05713-z.

## Background

Owing to its high incidence and low probability of survival and favorable neurological outcomes, out-of-hospital cardiac arrest (OHCA) is one of the major challenges of the healthcare system [1, 2]. In recent decades, scientific evaluations have focused on the optimal performance of chest compressions (CCs) to maintain blood flow and improvements in early defibrillation [2, 3].

Although intra-arrest ventilation has been recommended for decades to maintain adequate alveolar tidal volume despite the presence of dead space, most research has concentrated on optimal airway management strategies minimizing dead space, through tracheal intubation (ET) or various supraglottic airway devices (SGA), such as laryngeal tubes or i-gel laryngeal masks. These approaches remain subject to controversial discussions [2–9].

Portable emergency respirators are now also widely available internationally, enabling the use of differentiated ventilation strategies at the scene of an emergency. Despite these advances, manual bag-valve-device ventilation (BVDV) is a frequently used procedure in intra-arrest ventilation, especially during the initial phase of CPR performed by paramedics, and has undergone few changes since its introduction almost 70 years ago [10, 11].

Manual BVDV is inexpensive, lightweight, requires minimal maintenance, and can be applied under diverse environmental conditions. Its perceived advantages are ease of use and rapid availability. However, major limitations include operator dependency; lack of validated monitoring (e.g., tidal volumes, capnography); high demand for personal in resource-constrained situations; and risk of hyper- and hypoventilation as well as intrathoracic pressure peaks [7, 9–14].

However, due to the lack of monitoring systems, few data are available for both the applied volumes and the intrathoracic ventilation pressures of patients on BVDV. Therefore, the effects of different airway devices on the target parameters of ventilation therapy during BVDV were investigated in this prospective human cadaver study.

## Methods

This study on human cadavers was conducted between March and August 2024 at the Institute of Anatomy of Ruhr University Bochum after a positive vote of the ethics committee of Ruhr University Bochum in Ostwestfalen-Lippe, Bad Oeynhausen, Germany, on February 13, 2024 (Ref. 2024−1183) and registration at ClinicalTrials.gov (registration number NCT06306898). The body donors derived from the donor program of Ruhr University Bochum in collaboration with “Klinisch-Anatomisches Forschungs- und Fortbildungszentrum” (KAFFZ), Institute of Anatomy, Ruhr University Bochum, Germany. Owing to the limited availability of Thiel-embalmed cadavers, chest compression synchronized ventilation (CCSV) and intermittent positive pressure ventilation (IPPV) were examined on the same cadavers in addition to the BVDV, and the examinations were combined in one study registration [15]. However, the data collected in this way were analyzed prospectively and separately for each of the individual ventilation modes. The study was conducted in compliance with the ethical principles of the Declaration of Helsinki [16]. The structure of the paper is based on the current CONSORT guidelines [17].

### Inclusion and exclusion criteria

Adult Thiel-embalmed human cadavers were included [18]. Cadavers with abnormal airways, tracheostomies, premortem acute respiratory distress syndrome (ARDS) and severe lung or thoracic injuries, such as pneumothorax or severe aspiration, were excluded.

### Study design

**Fig. 1:**
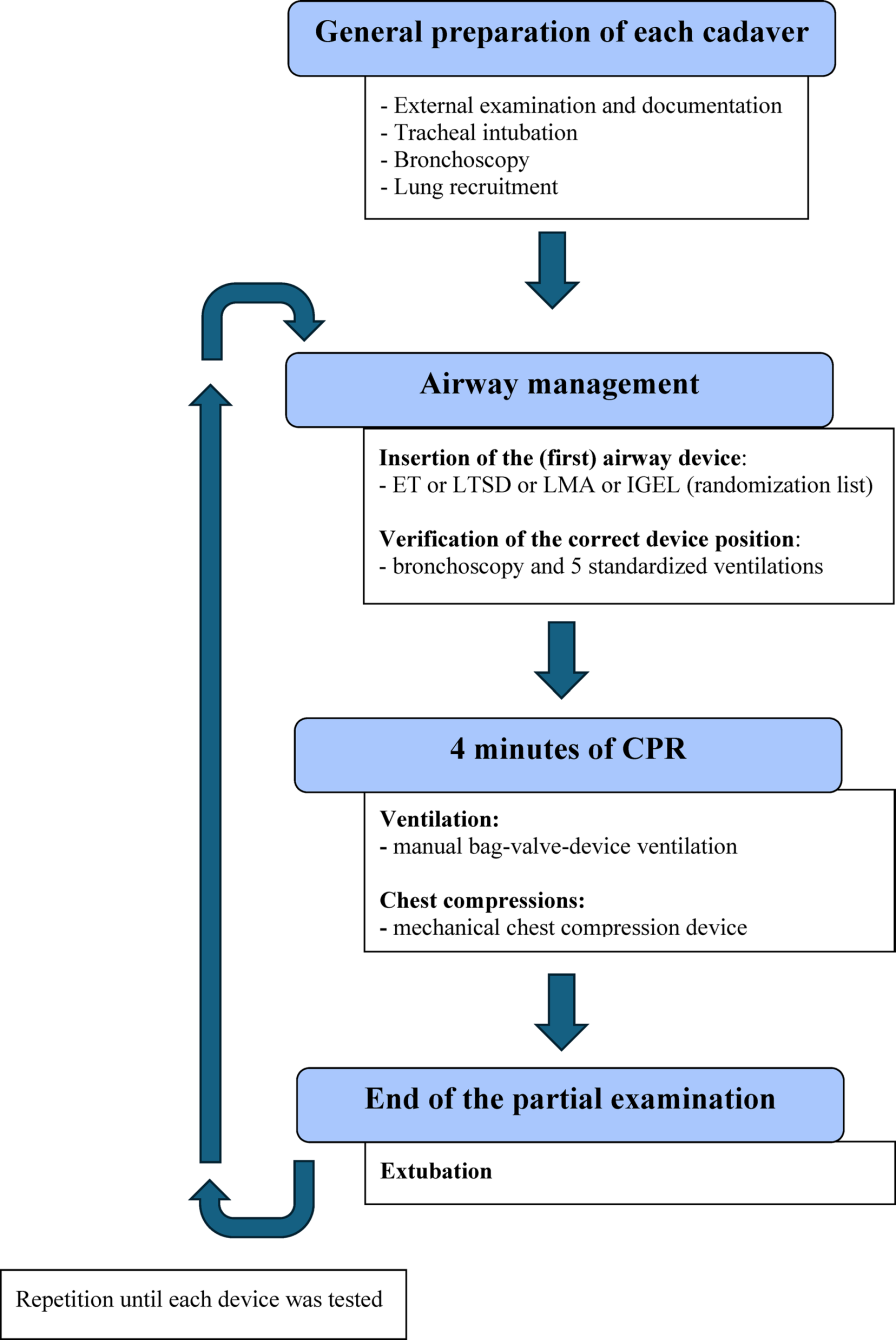
Flow chart of the experimental procedure

Each human cadaver underwent ventilation with four different airway devices, applied sequentially for four minutes each, during ongoing chest compressions. A schematic overview of the experimental procedure is provided in Fig. 1. The primary aim was to compare BVDV using ET versus various SGAs.

### General preparation of the cadavers

After an external postmortem examination with documentation of the biological sex, actual body weight and size of the cadaver, the ideal body weight (IBW) was calculated using Broca’s formula [19]. This process was followed by tracheal intubation via Mallinckrodt tubes (male = 8.0 inner diameter (ID in mm), female = 7.0 ID, cuff pressure = 40 cmH_2_O) and bronchoscopy to check the tube position and remove secretions.

In preparation for the experiment, the lungs were recruited for four minutes by pressure-controlled ventilation (inspiratory pressure (P_Insp_) = 35 mbar, positive end-expiratory pressure (PEEP) = 12 mbar, respiratory rate = 10 min^−1^) via MEDUMAT Standard^2^ (WEINMANN Emergency, Medical Technology GmbH + Co. KG, Hamburg, Germany). After completion of recruitment, the ET was removed.

### Airway management

After the lung recruitment, various airway devices were inserted according to the predefined randomization list: (1) control group: tracheal tube (= ET), (2) laryngeal tube (= LTSD) (LTS-D, VBM Medizintechnik GmbH, Sulz am Necker, Germany), (3) laryngeal mask (= LMA) (Ambu AuraGain, Ambu GmbH, Bad Nauheim, Germany), (4) i-gel^®^ laryngeal mask (= IGEL) (i-gel, Intersurgical GmbH, Sankt Augustin, Germany). The size of the respective SGA was selected according to the manufacturer’s specifications. The cuff pressure was limited to 40 cmH_2_O for all the cuffable airway devices and was checked via a cuff pressure device after the insertion of ET, LMA and LTSD. After each change in the airway device, the position was checked by bronchoscopy, and the lungs were recruited again using five standardized ventilations via the MEDUTrigger (MEDUMAT Standard^2^, WEINMANN Emergency, Medical Technology GmbH + Co. KG, Hamburg, Germany) before manual BVDV was started.

### Ventilation and chest compressions

Ventilation was performed using a bag-valve (Ambu Spur II, Ambu GmbH, Bad Nauheim, Germany) for four minutes by paramedics with several years of experience in prehospital emergency medicine.

The CCs were performed in a standardized manner using the mechanical CC device Corpuls-CPR (GS Elektromedizinische Geräte G. Stemple GmbH, Kaufering, Germany; frequency = 100 min^−1^; depth = 5.5 cm) [3].

### Change of the airway device

After four minutes of intra-arrest ventilation, the airway device was removed, and the next airway device was inserted according to the randomization list. The procedure was repeated on the same cadaver until each of the four airway devices had been evaluated (see Fig. 1).

### Measurement of the ventilation parameters

During the experiments, the ventilation parameters were measured and recorded via MEDUMAT Standard^2^ (WEINMANN Emergency, Medical Technology GmbH + Co. KG, Hamburg, Germany). For this purpose, parts of the measurement tubing system, which are used in mechanical ventilation to measure ventilation pressures, flow, and CO_2_ (FlowCheck sensor and adapter, CO_2_ measurement tube adapter, pressure measurement tube), were used and installed between the bag-valve and the airway device. The pressure measurement tube was connected to the CO_2_ adapter (see supplement 1).

The following parameters were recorded: expiratory tidal volume (Vt_e_), inspiratory tidal volume (Vt_i_), leakage volume (V_leak_), peak pressure (P_peak_), mean pressure (P_mean_), and manual ventilation frequency (F_manual_).

The calculated parameters included: ∆Vt (∆Vt = Vt_ideal_–Vt_e_*)*, the ideal tidal volume (Vt_ideal_) (calculated by the MEDUMAT Standard^2^), Vt_e_ (Vt_e_/kgIBW) and Vt_i_ per kilogram of ideal body weight (Vt_i_/kgIBW). Supplement 2 provides a detailed overview of the recorded and calculated ventilation parameters.

### Outcomes

The primary endpoint was ∆Vt. The secondary endpoints included Vt_e_, Vt_i_, MV_e_, V_leak_, Vt_e_/kgIBW, Vt_i_/kgIBW, P_peak_, P_mean_, and F_manual_.

### Statistical analysis

Data were first analyzed descriptively according to the variable type: means and standard deviations for continuous variables and numbers and proportions for categorical variables. For the primary outcome, a linear regression model with a random intercept was applied to account for potential intracadaver correlations. For all secondary outcomes, we again used linear regression models with random intercepts. As effect measures for all analyses, we report regression coefficients (RCs) with 95% confidence intervals (95% CIs) and p-values if appropriate. A p-value ≤ 0.05 was considered statistically significant. All analyses were performed using the statistical software SAS 9.4^®^ (SAS Institute Inc., Cary, NC).

## Results

**Fig. 2:**
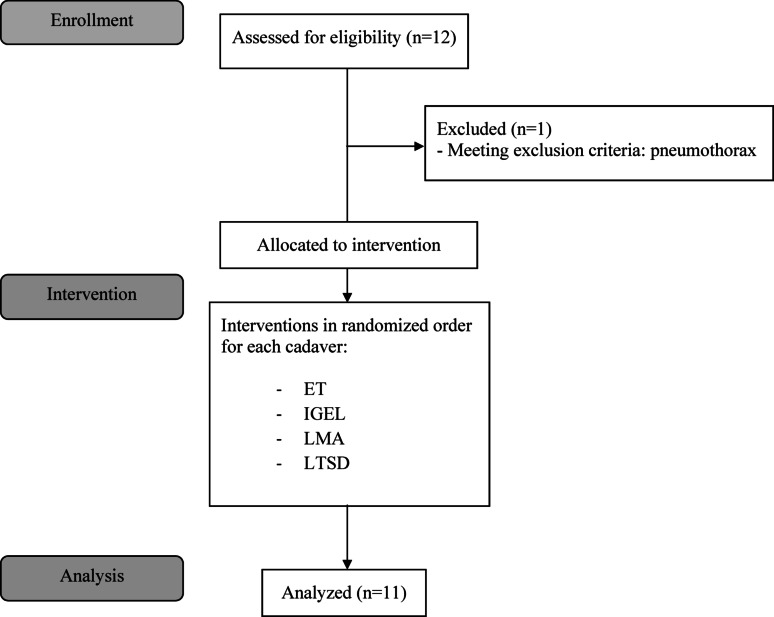
Flow chart of the study protocol (based on CONSORT 2025)

**Fig. 3:**
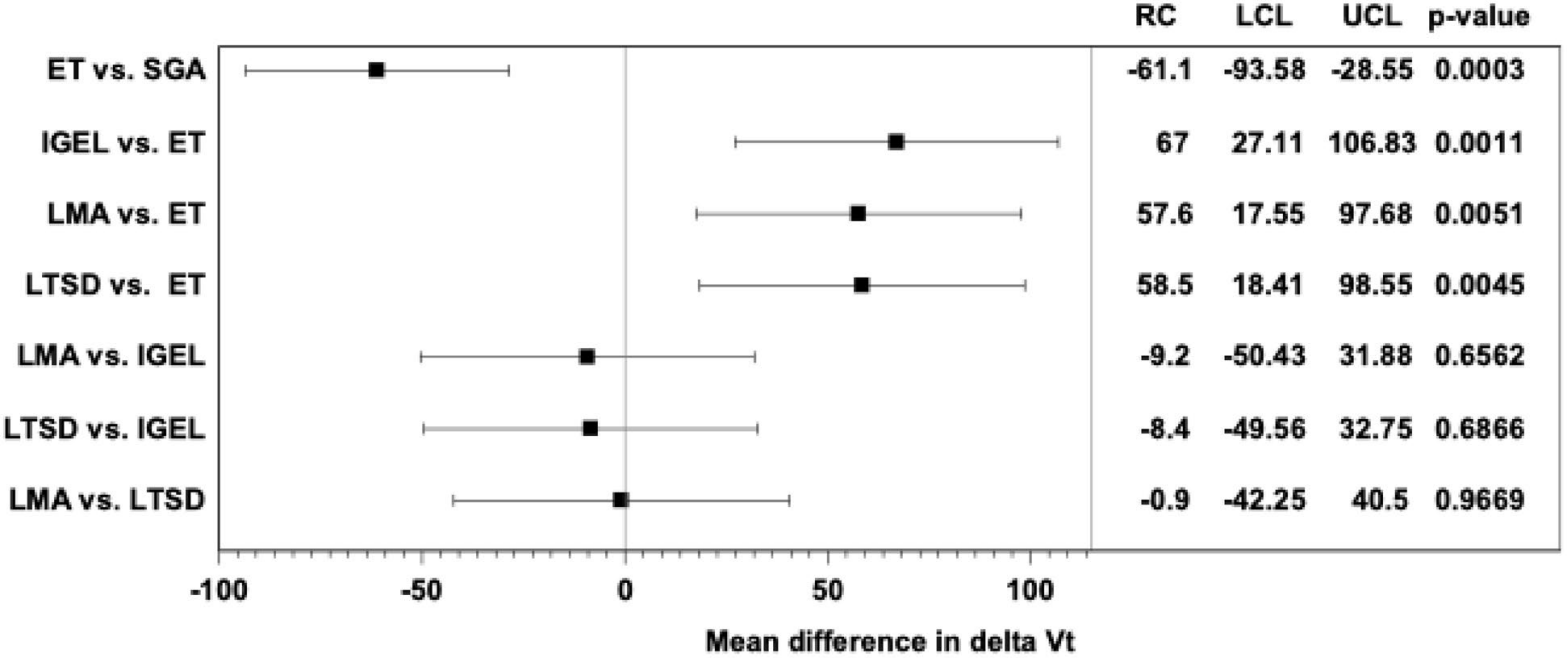
Results of the linear mixed regression models across the airway devices for the primary endpoint ∆Vt. Legend: ET = endotracheal tube, SGA = supraglottic airway devices, IGEL = i-gel laryngeal mask, LMA = laryngeal mask, LTSD = larnygeal tube, delta Vt = ∆Vt, RC = regression coefficient, LCL/UCL = lower control limit/upper control limit for 95% confidence interval

Out of a total of twelve human cadavers, eleven were included after applying all the inclusion and exclusion criteria. One cadaver was excluded because of an open pneumothorax (Fig. 2). Table 1 presents the characteristics of the included cadavers, while Table 2 provide descriptive statistics of the ventilation parameters according to the different airway devices used. Figure 3 presents the results of the linear mixed regression models across the airway devices for the primary endpoint ∆Vt.

**Table 1:** Characteristics of the included cadavers

No.	Sex (w/m)	Age (years)	Size (cm)	Body weight (kg)	Ideal body weight (kg)	Calculated alveolar dead space (2 ml/kg ideal body weight) (ml)	Ideal tidal volume (ml)	Body mass index (BMI)	Time death to fixation start (days)	Time fixation to experimental use (days)	Comorbidities
1	m	87	169	68	69	138	462	23.8	1	99	Benign prostate hyperplasia, coronary angioplasty
2	m	83	179	67	79	158	427	23.2	3	56	Bilroth II surgery
3	w	87	151	56	51	102	287	24.6	1	152	Heart and kidney failure, Coronary bypass
4	w	85	155	72	55	110	329	30.0	3	68	Breast surgery with silicone remodeling
5	m	83	172	70	72	144	462	23.7	1	90	None
6	m	83	179	75	79	158	525	23.4	1	22	None
7	w	91	158	64	58	116	190	25.6	1	136	Cholecystectomy, appendectomy, osteoporosis
8	w	81	178	75	78	156	366	23.7	1	76	None
9	m	83	164	57	64	128	366	21.2	3	63	Osteoarthrosis, abdominal aortic aneurysm
10	m	78	178	75	78	156	450	23.7	1	73	Uveal melanoma
11	m	81	168	75	68	136	396	26.6	2	27	Chronic obstructive pulmonary disease, osteoporosis, pancreatitis
Mean ± SD		83.8 ± 3.4	168.3 ± 9.7	68.5 ± 6.7	68.3 ± 9.7	136.5 ± 19.4	387.3 ± 89.9	24.9 ± 1.4	1.6 ± 0.9	78.4 ± 38.1	

**Table 2:** Descriptive statistics of the measured parameters across airway devices

Variable	ET (*n* = 44)	SGA (*n* = 133)	IGEL (*n* = 45)	LMA (*n* = 44)	LTSD (*n* = 44)
∆Vt (ml) [Mean ± SD]	142.2 ± 125.5	202.0 ± 153.1	205.6 ± 213.8	199.8 ± 116.2	200.6 ± 107.8
Vt_ideal_ (ml) [Mean ± SD]	387.3 ± 91.0	385.8 ± 91.6	382.9 ± 94.6	387.3 ± 91.0	387.3 ± 91.0
Vt_e_ (ml) [Mean ± SD]	245.1 ± 91.2	183.8 ± 122.1	177.3 ± 173.4	187.5 ± 83.3	186.6 ± 89.3
Vt_i_ (ml) [Mean ± SD]	322.9 ± 135.1	386.7 ± 127.6	382.7 ± 116.1	382.7 ± 132.2	394.7 ± 136.4
Vt_e_/kgIBW (ml/kg) [Mean ± SD]	3.6 ± 1.3	2.7 ± 1.7	2.7 ± 2.3	2.8 ± 1.2	2.8 ± 1.2
Vt_i_/kgIBW (ml) [Mean ± SD]	4.8 ± 2.1	5.7 ± 1.9	5.7 ± 1.8	5.7 ± 2.1	5.8 ± 2.0
V_leak_(%) [Mean ± SD]	17.9 ± 15.3	39.0 ± 23.5	38.4 ± 26.0	41.4 ± 19.5	37.4 ± 24.8
MV_e_ (l/min) [Mean ± SD]	3.5 ± 2.6	2.4 ± 1.8	2.1 ± 1.1	2.3 ± 1.4	2.7 ± 2.5
P_mean_ (mbar) [Mean ± SD]	4.0 ± 1.5	3.1 ± 1.0	3.0 ± 0.9	3.3 ± 1.1	3.2 ± 0.9
P_peak_ (mbar) [Mean ± SD]	47.7 ± 14.9	39.0 ± 10.0	41.5 ± 11.5	36.9 ± 6.5	38.6 ± 11.1
F_manual_ (x/min) [Mean ± SD]	9.2 ± 0.8	8.8 ± 2.0	8.4 ± 2.9	8.9 ± 1.6	9.1 ± 1.0

ET = endotracheal tube, SGA = supraglottic airway devices, IGEL = i-gel laryngeal mask, LMA = laryngeal mask, LTSD = laryngeal tube, ∆Vt = Vt_ideal_–Vt_e_, Vt_ideal_ = ideal tidal volume, Vt_e_ = exspiratory tidal volume, Vt_i_ = inspiratory tidal volume, Vt_e_/kgIBW = exspiratory tidal volume per kilogram ideal body weight, Vt_i_/kgIBW = inspiratory tidal volume per kilogram ideal body weight, V_leak_ =leckage volume, MV_e_ =exspiratory minute volume, P_mean_ = mean pressure, P_peak_ = peak pressure, F_manual_ = manual ventilation frequence, SD = standard deviation, ml = milliliter, kg = kilogram, l = liter, min = minute, mbar = millibar

Table 3 shows the results of the linear mixed regression models of various ventilation parameters between the ET and SGA groups. The comparison of the ET and SGA groups revealed lower ∆Vt (RC = –61.07 ml, 95% CI = [–93.58;–28.55], *p* = 0.0003), Vt_i_ (RC = –62.90 ml, 95% CI = [–80.25;–45.56], *p* < 0.0001), Vt_i_/kgIBW (RC = –0.91 ml, 95% CI = [–1.15;–0.66], *p* < 0.0001), and V_leak_ (RC = –20.98%, 95% CI = [–26.63;–15.33], *p* < 0.0001), but higher Vt_e_ (RC = 61.14 ml, 95% CI = [28.63;93.65], *p* = 0.0003), Vt_e_/kgIBW (RC = 0.88 ml, 95% CI = [0.44;1.32], *p* = 0.0001), P_mean_ (RC = 0.90 mbar, 95% CI = [0.59;–1.21], *p* < 0.0001), and P_peak_ (RC = 11.46 mbar, 95% CI = [8.65;14.26], *p* < 0.0001) in the ET group.

**Table 3:** Results of the linear mixed regression models for the comparison of tracheal intubation (reference group) and supraglottic airway devices

Variable	Regression coefficient	95% confidence interval	*p*-value
∆Vt (ml)	–61.07	[–93.58; −28.55]	0.0003
Vt_e_ (ml)	61.14	[28.63; 93.65]	0.0003
Vt_i_ (ml)	–62.90	[–80.25; −45.56]	< 0.0001
Vt_e_/kgIBW (ml/kg)	0.88	[0.44; 1.32]	0.0001
Vt_i_/kgIBW (ml/kg)	−0.91	[−1.15; −0.66]	< 0.0001
V_leak_ (%)	–20.98	[–26.63; −15.33]	< 0.0001
P_mean_ (mbar)	0.90	[0.59; 1.21]	< 0.0001
P_peak_ (mbar)	11.46	[8.65; 14.26]	< 0.0001

∆Vt = Vt_ideal_–Vt_e_, Vt_ideal_ = ideal tidal volume, Vt_e_ = exspiratory tidal volume, Vt_i_ = inspiratory tidal volume, Vt_e_/kgIBW = exspiratory tidal volume per kilogram ideal body weight, Vt_i_/kgIBW = inspiratory tidal volume per kilogram ideal body weight, V_leak_ = leckage volume, MV_e_ = exspiratory minute volume, P_mean_ = mean pressure, P_peak_ = peak pressure, F_manua l_ = manual ventilation frequence, SD = standard deviation, ml = milliliter, kg = kilogram, l = liter, min = minute, mbar = millibar

Table 4 shows the results of the linear mixed regression models of various ventilation parameters for the comparison of ET and different airway devices in the SGA group. In this comparison, all devices in the SGA group presented higher ∆Vt values (IGEL: RC = 66.97 ml, 95% CI = [27.11;106.83], *p* = 0.0011; LMA: RC = 57.61 ml, 95% CI = [17.55;97.68], *p* = 0.0051; LTSD: RC = 58.48 ml, 95% CI = [18.41;98.55], *p* = 0.0045) and lower Vt_e_ values (IGEL: RC=–67.19 ml, 95% CI = [–107.04;–27.33], *p* = 0.0011; LMA: RC = –57.61 ml, 95% CI = [–97.68;–17.55], *p* = 0.0051; LTSD: RC = –58.48 ml, 95% CI = [–98.55;–18.41], *p* = 0.0045) when directly compared with those in the ET group.

**Table 4:** Results of the linear mixed regression models for the comparison of tracheal intubation with different supraglottic airway devices

Variable	Regression coefficient	95% confidence interval	*p*-value
∆Vt (ml)			
IGEL vs. ET	66.97	[27.11; 106.83]	0.0011
LMA vs. ET	57.61	[17.55; 97.68]	0.0051
LTSD vs. ET	58.48	[18.41; 98.55]	0.0045
Vt_e_ (ml)			
IGEL vs. ET	–67.19	[–107.04; −27.33]	0.0011
LMA vs. ET	–57.61	[–97.68; −17.55]	0.0051
LTSD vs. ET	–58.48	[–98.55; −18.41]	0.0045
Vt_i_ (ml)			
IGEL vs. ET	57.21	[36.06; 78.36]	< 0.0001
LMA vs. ET	59.80	[38.54; 81.05]	< 0.0001
LTSD vs. ET	71.82	[50.56; 93.08]	< 0.0001
Vt_e_/kgIBW (ml/kg)			
IGEL vs. ET	–0.93	[–1.47; −0.39]	0.0008
LMA vs. ET	–0.84	[–1.38; −0.30]	0.0026
LTSD vs. ET	–0.87	[–1.41; −0.33]	0.0019
Vt_i_/kgIBW (ml/kg)			
IGEL vs. ET	0.81	[0.51; 1.11]	< 0.0001
LMA vs. ET	0.90	[0.60; 1.20]	< 0.0001
LTSD vs. ET	1.01	[0.71; 1.31]	< 0.0001
V_leak_ (%)			
IGEL vs. ET	19.93	[13.03; 26.83]	< 0.0001
LMA vs. ET	23.53	[16.59; 30.46]	< 0.0001
LTSD vs. ET	19.50	[12.56; 26.44]	< 0.0001
P_mean_ (mbar)			
IGEL vs. ET	–1.05	[–1.43; −0.67]	< 0.0001
LMA vs. ET	–0.78	[–1.15; −0.40]	< 0.0001
LTSD vs. ET	–0.88	[–1.26; −0.50]	< 0.0001
P_peak_ (mbar)			
IGEL vs. ET	–8.98	[–12.34; −5.62]	< 0.0001
LMA vs. ET	–13.54	[–16.91; −10.18]	< 0.0001
LTSD vs. ET	–11.88	[–15.24; −8.51]	< 0.0001

ET = endotracheal tube, SGA = supraglottic airway devices, IGEL = i-gel laryngeal mask, LMA = laryngeal mask, LTSD = laryngeal tube, ∆Vt = Vt_ideal_–Vt_e_, Vt_ideal_ = ideal tidal volume, Vt_e_ = exspiratory tidal volume, Vt_i_ = inspiratory tidalvolume, Vt_e_/kgIBW = exspiratory tidal volume per kilogram ideal body weight, Vt_i_/kgIBW = inspiratory tidal volume per kilogram ideal body weight, V_leak_ = leckage volume, MV_e_ = exspiratory minute volume, P_mean_ = mean pressure, P_peak_ = peak pressure, F_manual_ = manual ventilation frequence, SD = standard deviation, ml = milliliter, kg = kilogram, l = liter, min = minute, mbar = millibar

Table 5 shows the results of the linear mixed regression models for the comparison of various ventilation parameters for SGA. Except for P_peak_ (RC = –4.56 mbar, 95% CI = [–7.47;–1.66], *p* = 0.0025) in the comparison of LMA and IGEL, no evidence for further differences were observed.

**Table 5:** Results of the linear mixed regression models for the comparison of the different supraglottic airway devices

Variable	Regression coefficient	95% confidence interval	*p*-value
∆Vt (ml)			
LMA vs. IGEL	–9.23	[–50.43; 31.88]	0.6562
LTSD vs. IGEL	–8.41	[–49.56; 32.75]	0.6866
LMA vs. LTSD	–0.87	[–42.25; 40.50]	0.9669
Vt_e_ (ml)			
LMA vs. IGEL	9.53	[–31.62; 50.69]	0.6473
LTSD vs. IGEL	8.66	[–32.49; 49.82]	0.6775
LMA vs. LTSD	0.87	[–40.50; 42.24]	0.9669
Vt_i_ (ml)			
LMA vs. IGEL	2.59	[–17.43; 22.62]	0.7980
LTSD vs. IGEL	14.62	[–5.40; 34.64]	0.1509
LMA vs. LTSD	–12.03	[–32.15; 8.10]	0.2392
Vt_e_/kgIBW (ml/kg)			
LMA vs. IGEL	0.09	[–0.45; 0.63]	0.7404
LTSD vs. IGEL	0.06	[–0.48; 0.60]	0.8205
LMA vs. LTSD	0.03	[–0.51; 0.57]	0.9172
Vt_i_/kgIBW (ml/kg)			
LMA vs. IGEL	0.09	[−0.20; 0.38]	0.5412
LTSD vs. IGEL	0.20	[−0.09; 0.49]	0.1693
LMA vs. LTSD	−0.11	[−0.40; 0.18]	0.4452
V_leak_ (%)			
LMA vs. IGEL	3.53	[–4.10; 11.16]	0.3617
LTSD vs. IGEL	–0.49	[–8.13; 7.14]	0.8982
LMA vs. LTSD	4.03	[–3.65; 11.70]	0.3012
P_mean_ (mbar)			
LMA vs. IGEL	0.27	[–0.02; 0.57]	0.0712
LTSD vs. IGEL	0.17	[–0.12; 0.46]	0.2534
LMA vs. LTSD	0.10	[–0.19; 0.39]	0.5001
P_peak_ (mbar)			
LMA vs. IGEL	–4.56	[–7.47; −1.66]	0.0025
LTSD vs. IGEL	–2.90	[–5.81; 0.01]	0.0507
LMA vs. LTSD	–1.67	[–4.57; 1.24]	0.2577

ET = endotracheal tube, SGA = supraglottic airway devices, IGEL = i-gel laryngeal mask, LMA = laryngeal mask, LTSD = laryngeal tube, ∆Vt = Vt_ideal_–Vt_e_, Vt_ideal_ = ideal tidal volume, Vt_e_ = exspiratory tidal volume, Vt_i_ = inspiratory tidal volume, Vt_e_/kgIBW = exspiratory tidal volume per kilogram ideal body weight, Vt_i_/kgIBW = inspiratory tidal volume per kilogram ideal body weight, V_leak_ = leckage volume, MV_e_ = exspiratory minute volume, P_mean_ = mean pressure, P_peak_ = peak pressure, F_manual_ = manual ventilation frequence, SD = standard deviation, ml = milliliter, kg = kilogram, l = liter, min = minute, mbar = millibar

## Discussion

### Main findings

This study investigated the influence of different airway devices on the ∆Vt of human cadavers during BVDV. The comparison among the SGA group as a whole and individual devices in the SGA group with the ET group revealed a lower ∆Vt and V_leak_ as well as a higher P_peak_. When individual devices in the SGA group were compared with each other, there was no evidence for differences between them, apart from the lower P_peak_ for LMA vs. IGEL.

### Importance of tidal volume

Although intra-arrest ventilation has been recommended as part of CPR for decades, its optimal implementation has been much less scientifically evaluated than other CPR measures. After successful airway management, the resuscitation guidelines currently recommend ventilation at a rate of 10 min^−1^ with continuous CCs or, if a leak in SGA leads to inadequate ventilation, at a ratio of 30:2, as well as the application of a tidal volume (Vt) of 500–600 ml or 6–8 ml/kgIBW [2, 3]. Although the corresponding Vt following the recommendations can be set on the respirator as part of mechanical ventilation, studies have shown that for intra-arrest ventilation, regardless of the type of respirator used, the Vt actually applied sometimes deviated significantly from the set Vt [20–23]. While mechanical ventilation is a common part of resuscitation protocols in European emergency medical services, BVDV is widely used in the United States of America. Although studies have shown that both hyperventilation and hypoventilation during CPR is common with BVDV and is associated with an unfavorable impact on prognosis, its proponents argue that intra-arrest ventilation increases the ability of the lungs to be ventilated and avoids inadequate Vt or pressure peaks [7, 24–26]. In contrast, however, the data collected from human cadavers as part of the present study demonstrated significant deviations between the ideal and applied Vt during intra-arrest ventilation via BVDV, analogous to mechanical ventilation, particularly when a SGA was used. The possible causes are as follows: first, airway leakage due to accidental dislocation of the SGA used during the dynamic situation of cardiopulmonary resuscitation (CPR); second, increased intrathoracic pressures due to CC counteraction of the pressure gradient required to apply sufficient tidal volumes; third, necessary application of higher ventilation pressures to compensate for the increased intrathoracic pressures caused by CCs also favored pronounced leakage volumes, especially when SGA was used. In the present study, the lowest ∆Vt and the highest Vt_e_/kgIBW were observed when ET was used. These findings suggest that this device could be beneficial in terms of applied Vt under continuous CCs.

### Importance of ventilation pressure

The application of positive pressures is the basis of positive pressure ventilation. Although ventilation and its influence on organ systems and functions of ventilated patients have been well studied in the clinical context, the effects of applied ventilation volumes and pressures in intra-arrest ventilation are not yet fully understood. Studies have shown effects on both gas exchange and hemodynamics [7, 12, 14, 27]. Although there is evidence that compression-synchronized cyclic changes in intrathoracic pressure may lead to an increase in mean arterial pressure and thus improve resuscitation efforts, other studies indicate a possible compromise of the minimal circulation by an increase in mean intrathoracic pressure [12, 24]. From the perspective of lung-protective ventilation to prevent barotrauma and secondary inflammatory changes (ARDS and manual-ventilation-induced lung injury (MVILI)), the use of the lowest possible peak pressures also appears plausible, although the current resuscitation guidelines recommend values between 60 − 70 cmH_2_O as upper limits for peak pressures for the IPPV [2, 3, 10, 28]. In the present study, ventilation with ET was associated with higher ventilation pressures (P_mean_ and P_peak_) than ventilation with BVDV via SGA, mainly because of the lower leakage with an endotracheal blocked tube [29–32]. While this phenomenon may have a positive effect on ET due to increased tidal volumes with respect to the respiratory aspect of CPR, the hemodynamic risks of increased intrathoracic pressure and the occurrence of barotrauma are potential risks of BVDV. Furthermore, increased intra-abdominal pressures due to gastric air insufflation, with the risk of regurgitation, aspiration and negative effects on hemodynamics, are also described as complications when SGA is used owing to the increased leakage volume [12, 32–34].

Although the effects of different ventilation pressure levels are not yet fully understood, the data from the present study indicate relevant differences in the BVDV between the different airway devices used. These factors may also have influenced previous studies evaluating the impact of different airway devices on the prognosis of patients with OHCA [4–6, 29, 35]. Although there are sometimes significant differences between the various airway devices, not only in terms of design but also in terms of the efficacy of the BVDV, virtually none of these studies adjusted for the ventilation strategy used, which represents a relevant bias that has rarely been addressed to date. Future studies are needed to evaluate the effects of different ventilation modes and pressure levels when comparing different airway devices in real resuscitation situations.

### Importance of BVDV in intra-arrest ventilation

BVDV is a frequently used procedure in intra-arrest ventilation, especially in the initial phase of CPR [10]. Conversely, the efficacy and safety of the procedure are heavily dependent on the user, as validated monitoring of the applied ventilation volumes, pressures, frequencies and often capnography for intra-arrest ventilation is rarely available as a standard for BVDV. Although guideline-compliant use of BVDV appears non-inferior to mechanical ventilation in terms of the rate of return of spontaneous circulation (ROSC), several studies emphasize the high prevalence of undetected hyper- and hypoventilation and their adverse effects [24–26, 36]. Regardless of the airway device or ventilation mode used, effective ventilation requires adequate alveolar delivery. The applied tidal volume must exceed the dead space and compensate for potential leakage volumes, which may occur when using SGA in particular but not exclusively, without generating intrathoracic pressure that compromises blood flow.

Considering the importance of sufficient tidal volumes at the lowest possible ventilation pressures during BVDV and the potentially associated negative effects on CPR, the current procedure without feedback systems for monitoring ventilation therapy appears to be extremely questionable in this context. Instead, the authors consider monitoring the relevant ventilation parameters during BVDV necessary, ideally in real time. On the one hand, this would make it possible to objectify the effectiveness of ventilation during BVDV, regardless of the device used, and in particular to switch from asynchronous ventilation with continuous CCs to resuscitation at a ratio of 30:2 in the event of ineffective ventilation (e.g., via the SGA) and to adapt this to the patient in terms of individualized ventilation. On the other hand, from a scientific perspective, this could lead to a better understanding of the special features of BVDV during intra-arrest ventilation in the future and thus to better comparability with mechanical ventilation, with the aim of individualized optimal intra-arrest ventilation. Taken together, this experimental study represents only an initial step toward improving intra-arrest ventilation in OHCA. Further studies are urgently needed to optimize intra-arrest ventilation.

### Limitations

This study has several limitations that should be considered when interpreting the findings. One is the limited sample size, which may limit the significance of the results and their generalizability. Nevertheless, the available data yielded statistically significant results that indicate sufficient statistical power. Another aspect concerns the Thiel embalming method: the tissue of the cadavers could differ from that of real resuscitation patients owing to the effects of this fixation. However, in view of insufficiently established measurement methods for the valid determination of ventilation parameters during BVDV/intra-arrest ventilation under real operating conditions, Thiel´s embalming method - due to the preservation of tissue elasticity and anatomical integrity - is currently one of the most accurate models for anatomical and surgical trainings. The limitation of the duration of resuscitation to four minutes allows only limited validity in comparison with that of resuscitation measures, which are sometimes necessary for much longer durations. Owing to the limited availability of Thiel-embalmed cadavers, CCSV and IPPV were examined on the same cadavers in addition to BVDV. Each ventilation mode was evaluated with each airway device for a period of four minutes, resulting in a total ventilation time under CCs of 48 min for each cadaver, which may have had a decisive impact on the integrity and viscoelastic properties of the tissue and therefore potentially influenced the parameters measured. In the present study, both the ventilation patterns used, and the airway devices were randomized to ensure that the possible effects of continuous resuscitation time on the body donors were evenly distributed. However, this is frequently prolonged in prehospital setting. Since Vt_ideal_ is fixed, the primary endpoint ∆Vt is largely driven by Vt_e_ and influenced by V_leak_, which could have also been considered a primary endpoint, as it directly affects Vt_e_. Furthermore, because ventilation was performed manually, variability in Vt_i_ between cycles could also impact Vt_e_. However, from a clinical-practical perspective, it was useful to choose this endpoint to give prehospital emergency physicians a pragmatically understandable impression of the effects of the various airway devices on the volumes administered during intra-arrest ventilation with BVDV. Due to the lack of evaluated ventilators for assessing manual ventilation, the MEDUMAT Standard^2^ was used - originally designed for measuring mechanical ventilation. Furthermore, the use of different airway devices may have influenced the ventilation conditions for subsequent airway devices, e.g., due to alveolar collapse. However, the order of the airway devices was randomized, and after each new insertion, the lungs were recruited again.

Despite these limitations, this study provides valuable insights into the effects of different airway devices during intra-arrest ventilation in a randomized controlled human cadaver model.

## Conclusions

Compared with the different SGAs, intra-arrest ventilation via the BVDV under continuous CCs using ET resulted in lower ∆Vt and V_leak_ values as well as higher P_peak_ values, whereas there was no evidence for differences in ∆Vt when the SGAs were compared with each other. Future studies should investigate the influence of ventilation monitoring in the context of BVDV and its effects on outcomes.

## Supplementary Information

Supplementary Material 1

## Data Availability

The datasets used and/or analyzed during the current study are available from the corresponding author on reasonable request.

## Abbreviations

ARDS: Acute respiratory distress syndrome
BVDV: Bag-valve-device ventilation
CC(s): Chest compression(s)
CCSV: Chest compression synchronized ventilation
CI: Confidence interval
CPR: Cardiopulmonary resuscitation
∆Vt: Difference between Vt_ideal_ and Vt_e_ (Vt_ideal_–Vt_e_)
ET: Tracheal tube
F_manual_: Manual ventilation frequency
IBW: Ideal body weight
ID: Inner diameter
IGEL: i-gel^®^ laryngeal mask
IPPV: Intermittent positive pressure ventilation
LMA: Laryngeal mask
LTSD: Laryngeal tube
MVILI: Manual-ventilation-induced lung injury
OHCA: Out-of-hospital cardiac arrest
PaCO_2_: Partial pressure of arterial carbon dioxide
PaO_2_: Partial pressure of arterial oxygen
PEEP: Positive end-expiratory pressure
P_Insp_: Inspiratory pressure
P_mean_: Mean pressure
P_peak_: Peak pressure
RC: Regression coefficient
ROSC: Return of spontaneous circulation
SGA: Supraglottic airway device
V_leak_: Leakage volume
Vt: Tidal volume
Vt_e_: Expiratory tidal volume
Vt_e_/kgIBW: Vt_e_ per kilogram of ideal body weight
Vt_i_: Inspiratory tidal volume
Vt_i_/kgIBW: Vt_i_ per kilogram of ideal body weight
Vt_ideal_: Ideal tidal volume
