# Supplementary material for: Influence of different airway devices on intra-arrest ventilation during bag-valve-device ventilation - a prospective randomized controlled cadaver study
Source: Crit Care. 2025 Dec 5;29:519. doi: 10.1186/s13054-025-05713-z (PMC12681136; doi:10.1186/s13054-025-05713-z)
Supplement: Supplementary file 1 — Supplementary Material 1 [file 13054_2025_5713_MOESM1_ESM.docx]

**
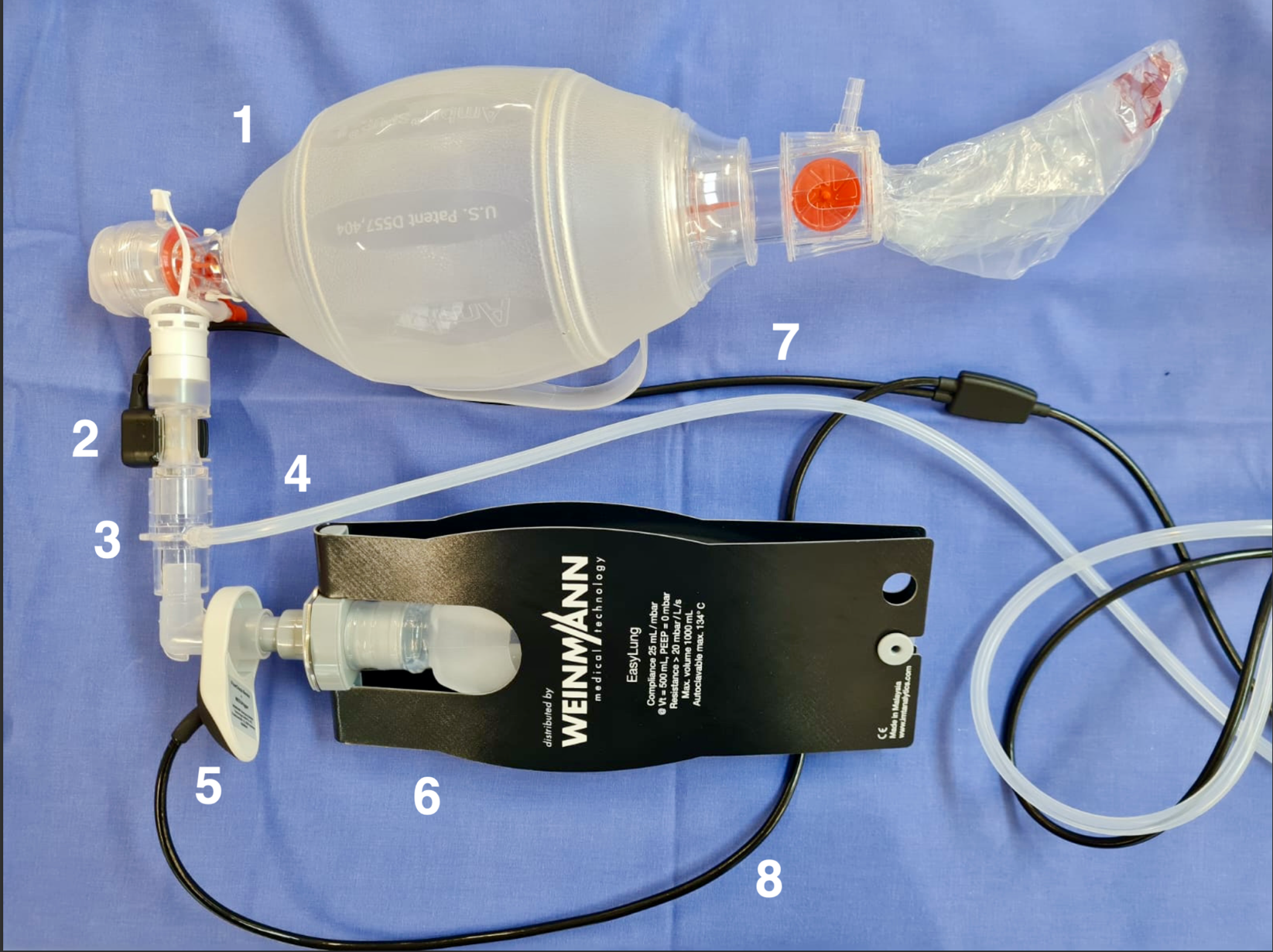
Supplement 1 – Figure of the airway pressure measurement**

Legend: 1 - disposable resuscitator^1^, 2 - FlowCheck sensor^2^, 3 – connector for CO_2_ measuring tube^2^ ,4 – pressure measuring tube^2^, 5 – MEDUtrigger^2^, 6 - testing bag^2^ (as visualization of connected device), 7 – FlowCheck sensor connection line^2^, 8 – MEDUtrigger connection line^2^

^1^ Ambu Spur II, Ambu GmbH

^2^ WEINMANN Emergency, Medical Technology GmbH + Co. KG

**Supplement 2 – Table of measured ventilation parameters and calculation methods**

| **Parameters** | **Description** | **Calculation** |
| --- | --- | --- |
| **∆Vt** (ml) | Parameter for visualizing effective ventilation | ∆Vt = Vt_ideal_–Vt_e_ |
| **Vt_ideal_** (ml) | Calculated tidal volume based on the ideal body weight (IBW) | Calculated by the mechanical ventilator:  IBW female (kg) = 45 + 2.3 x (height in cm/2.54−60)  IBW male (kg) = 50 + 2.3 x (height in cm/2.54−60)  multiplicated with 6 ml |
| **Vt_e_ (**ml) | Expiratory tidal volume | Mean value over the last 5 expiratory volumes. Vt_e_ (single) calculates as integral over the expiration  (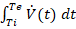 with Ti as begin and Te as end of the expiration).  Measured by the mechanical ventilator. |
| **Vt_i_** (ml) | Inspiratory tidal volume | Mean value over the last 5 inspiratory volumes. Vt_i_ (single) calculates as integral over all positive flows in one breath  (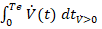with 0 as start of breath and Te as end).  Measured by the mechanical ventilator. |
| **V_leak_** (%) | Relative leakage volume | 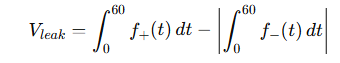Integral of the flows over one minute (positive flows - negative flows) divided by the inspiratory minute volume.  Measured by the mechanical ventilator. |
| **MV_e_ (**l/min) | Expiratory volume per minute | Minute average of the expiratory tidal volume measured by the device.  MVe=Vt×F, averaged over 5 breaths |
| **P_mean_** (mbar) | Mean pressure | Average value of the pressure over one minute. All pressure values measured by the mechanical ventilator during a breath were totaled and divided by the number of time intervals in which the measurement was taken. |
| **P_peak_** (mbar) | Peak pressure | Mean value of the maximum airway pressure (peak pressure) per respiratory cycle over 1 minute.  Measured by the mechanical ventilator. |
| **F_manual_** (x/min) | Manual ventilation frequency | Trend value measured by the device, averaged over 5 breaths. |
